# Verbascoside promotes apoptosis by regulating HIPK2–p53 signaling in human colorectal cancer

**DOI:** 10.1186/1471-2407-14-747

**Published:** 2014-10-05

**Authors:** Lihong Zhou, Yuanyuan Feng, Yongjie Jin, Xuan Liu, Hua Sui, Ni Chai, Xingzhu Chen, Ningning Liu, Qing Ji, Yan Wang, Qi Li

**Affiliations:** Department of Medical Oncology, Shuguang Hospital, Shanghai University of Traditional Chinese Medicine, Shanghai, 201203 China; Cancer Institute and Longhua Hospital, Shanghai University of Traditional Chinese Medicine, Shanghai, 200032 China

**Keywords:** Verbascoside, Homeodomain Interacting Protein Kinase 2, p53, apoptosis, colorectal cancer

## Abstract

**Background:**

We investigated the role of the HIPK2–p53 signaling pathway in tumorigenesis and resistance to the drug Verbascoside (VB) in colorectal cancer (CRC), using *in vivo* and *in vitro* experiments.

**Methods:**

Primary human CRC samples and normal intestinal tissues from patients were analyzed for HIPK2 expression by immunohistochemistry (IHC) and its expression was correlated against patients’ clinicopathological characteristics. Human CRC HCT-116 cells were implanted in BALB/c nude mice; mice with xenografted tumors were randomly administrated vehicle (control), 20, 40, or 80 mg/mL VB, or 1 mg/mL fluorouracil (5-FU). HIPK2, p53, Bax, and Bcl-2 expression in these tumors were determined by IHC. I*n vitro* effects of VB on CRC cell proliferation and apoptosis were measured by CCK-8 assay and flow cytometry; HIPK2, p53, p-p53, Bax, and Bcl-2 were measured by western blot.

**Results:**

IHC analysis for 100 human CRC tumor samples and 20 normal intestinal tissues, showed HIPK2 expression to inversely correlate with Dukes stage and depth of invasion in CRC (*P* < 0.05). *In vivo*, the inhibition rates of 20, 40, and 80 mg/mL VB on CRC xenograft tumor weight were 42.79%, 53.90%, and 60.99%, respectively, and were accompanied by increased expression of HIPK2, p53, and Bax, and decreased Bcl-2 expression in treated tumors. *In vitro*, VB significantly inhibited proliferation of CRC cell lines HCT-116, HT-29, LoVo, and SW620, in a time- and dose-dependent manner. The apoptosis rates of 25, 50, and 100 μM VB on HCT-116 cells were 10.83 ± 1.28, 11.25 ± 1.54, and 20.19 ± 2.87%, and on HT-29 cells were 18.92 ± 6.12, 21.57 ± 4.05, and 25.14 ± 6.73%, respectively. In summary, VB treatment significantly enhanced the protein expression of pro-apoptotic HIPK2, p53, p-p53, Bax, and decreased anti-apoptotic Bcl-2 expression in CRC cells.

**Conclusions:**

HIPK2 protein modulates the phosphorylation status of p53, and levels of Bax and Bcl-2 in CRC. We also found that VB effectively activated the HIPK2–p53 signaling pathway, resulting in increased CRC cell apoptosis.

## Background

Colorectal cancer (CRC) is one of the most common malignancies in the world. With economic development and lifestyle changes, the incidence of CRC has been increasing yearly, with a significant rising rate. According to Global Cancer Statistics 2011, the incidence of CRC ranked third among male cancer patients and second among female cancer patients. In 2011, people who died from CRC accounted for 8% of all cancer deaths. It is the fourth most common cause of cancer death [1]. In China, the rate of CRC incidence is increasing faster nationally than all other cancers. In the Shanghai area, CRC went from the fourth most common cancer in 1980s to the third most common in the 1990s [2] and by 2009 had become the second most common cancer in Shanghai [3].

Various factors contribute to CRC development, including intestinal mucosa losing normal growth control at the genetic level, leading to cell hyperproliferation. Most recent investigations of CRC tumorigenesis have therefore focused on functional abnormalities of relevant genes and their products.

Homeodomain Interacting Protein Kinase 2 (HIPK2) is a member of the serine/threonineproteinkinase family, located inside the cell nucleus. It is a transcription mediator that interacts with homobox plastein. Reportedly, HIPK2 is associated with late embryogenesis, and neural, retinal, and muscle tissue development, and also participates in various aspects of tumorigenesis, including oncogene expression [4], apoptosis [5], angiogenesis [6], and multi-drug resistance [7–9].

HIPK2 is a key regulator of numerous transcription factors, including p53, in DNA damage signaling pathways. HIPK2 co-localizes with p53in nucleosomes and phosphorylates Ser46 of p53. Using a microarray assay, Puca et al. found that *HIPK2* knockdown in colon cancer cells led to the loss of target gene activation of wild-type *p53* [10]. They also identified misfolding of p53 protein, and impaired p53–DNA binding and transcription of target genes. HIPK2 stimulates p300 and lys382-p53 for co-recruitment onto apoptosis promoters. By balancing p53 acetylation and deacetylation, HIPK2 regulates p53 apoptosis-promoting transcription activity [11]. McDonough et al. found HIPK2interacts with DAXX, a p53-binding protein, to inhibit binding with downstream effect or proteins, thus activating Ser46 phosphorylation and promoting p53 apoptotic signaling [12].

Verbascoside (VB), an active constituent of a Chinese traditional medical plant genus, *Cistanche*, has been shown to have anti-cancer activity in treating CRC, stomach [13], breast [14, 15], prostate [16], melanoma [17], glioma [18], and other cancers. Cistanche, as a common clinical treatment for CRC, inhibits post-operative tumor recurrence, tumor invasion and metastasis, although the underlining mechanisms are not yet well understood.

In this study, we analyzed HIPK2 expression in primary tumor specimens of human CRC, with particular regard to post-operative cancer recurrence, metastasis, and malignancy grades. We used a xenograft CRC mouse model to test the *in vivo* anti-tumor effect of VB and measured protein levels of HIPK2 and p53, and apoptosis-related gene products Bax and Bcl-2. We also show that VB inhibits cell proliferation and promotes apoptosis in CRC by stimulating the HIPK2–p53 signaling pathway.

## Methods

### Cell culture

Human CRC cell lines HCT-116, LoVo, HT-29, and SW62were purchased from the Chinese Academy of Science. HCT-116 and LoVo were cultured in RPMI-1640 medium with 10% fetal bovine serum (FBS), HT-29 and SW620 were cultured in McCoy’s 5A medium with 10% FBS. All cells were cultured with 100 μg/mL streptomycin (Invitrogen, Carlsbad, CA, USA) at 37°C in a 5% CO_2_ humidified incubator (Thermo Fisher Scientific Inc., Waltham, MA, USA).

### Human tissue samples

Human CRC tumor and normal tissue samples were collected from the General Surgery Department of our hospital from January 2011 to February 2012. All the experiments and animal care were approved by Shanghai Medical Experimental Animal Care Commission and in accordance with the Provision and General Recommendation of Chinese Experimental Animals Administration Legislation. The tissues were immediately frozen in liquid nitrogen and later preserved at ^−^80°C for long-term storage. The use of all human tissue samples was approved by the Institutional Review Board of the Shuguang Hospital affiliated to Shanghai University of Traditional Chinese Medicine. We obtained consent from every patient, for the use of all human tissues used in this study.

### Animals

BALB/c nude male mice, aged 4–6 weeks and weighing 18–20 g, were purchased from Sino-British SIPPR/BK lab Animal Co., Ltd (Shanghai, China, license No. SCXK 2008–0016). All animal protocols were approved by the Institutional Animal Use and Care Committee of Shanghai University of Traditional Chinese Medicine. Breeding conditions of the SPF Animal Laboratory were: free access to food and water, ventilation, humidity at 50–65%, temperature at 22–24°C, 12 h of light/dark. The animal laboratory abided by related regulations of the Animal Ethics Committee.

### Immunohistochemical staining

The human CRC tumor and normal tissue samples were paraffin embedded and serially sectioned. Tissue sections were processed by de-paraffining, rehydrating through an alcohol gradient, peroxidase clearing, antigen retrieval and blocking, antibody binding, DAB staining, washing with distilled water, hematoxylin staining, niacin alcohol differentiation, dilute ammonia bluing, incremental graded alcohol dehydration, xylene and conventional resin mounting. The primary antibody was rabbit-anti-human HIPK2 monoclonal antibody diluted by 1:50 (Abcam, Cambridge, MA, USA). The secondary biotin-labeled antibody was used at 1:200. For color development, streptavidin was labeled with horseradish peroxidase at 1:200. Under 400 × magnification, five random fields were selected. Staining was assessed as: non-staining: 0 point; light brown: 1 point; brownish yellow: 2 points; and dark brown: 3points. Percentages of positive-stained cells were rated as: positive cells ≤5%: 0 point; 6–25%: 1 point; 26–50%:2 points; and ≥75%: 3 points. Points for staining and percentage were multiplied for a 10-point scale: 0 point: negative (−), 1–3 points: weakly positive (+); 4–6 points: positive (++); and 7–9 points: strongly positive (+++).

### In vitro cell proliferation test

Human CRC HCT-116, LoVo, HT-29, and SW620 cells in logarithmic growth phases were plated at 5 × 10^3^ cells/well in 96-well plates; the next day, culture media was replaced with 200 μL culture medium containing VB (purity >98%, purchased from Chendu Herb purify Biotechnology Co., Ltd, Chendu, China, serial number: 20100123), with concentrations of 12.5, 25, 50, 100, 150, or 200 μM. For each concentration, 12 ventral orifices were set. After 24 h, 48 h, and 72 h, 20 μL of CCK-8 reagent (Dojindo Molecular Technologies, Inc., Tokyo, Japan) was added into each well. Four hours later, the light absorption value of each well at 490 nm was measured in a microplate reader (Bio-Rad Laboratories, Philadelphia, PA, USA). The inhibition rate of VB on the growth of CRC cells was calculated as the following equation: GIR = [1− (OD_N_ − OD_0_)/(OD_C_ − OD_0_) ] × 100%; where OD_0_ was the absorbance value of the blank group, OD_C_ the control group, and OD_N_ groups with different doses of VB. The IC_50_ of VB was calculated using three independent experiments.

### Apoptosis measured by flow cytometry

Rapid growing HCT-116 and HT-29 cells were treated with VBat different concentrations (25, 50, or 100 μM) for 48 h. Cells were then stained with 2 μL Annexin-V and 2 μL PI in 50 μL of apoptosis reaction solution at 4°C for 30 min. FACScan flow cytometry was used to detect apoptotic cells. Cell debris in different quadrants was calculated statistically. Cells in the upper right quadrant represented early apoptosis; cells in the lower right quadrant represented late apoptosis.

### In vivo xenografic CRC model

HCT-116 cells (2 × 10^6^/mouse) were injected subcutaneously into the right axilla of nude mice. Ten to 14 days later, when tumors formed, the nude mice with good growth state and unbroken tumors were used as tumor supply mice, and were then sacrificed. Tumors were dissected out under aseptic conditions, with necrotic and fibrous tissues removed. Fresh parts on the edge of tumors were cut into 1-mm^3^tumor blocks, which were implanted under the axillar skin of the right front legs of nude mice. With this method, three generations of mice were produced. The third-generation mice with unbroken transplanted tumor and sound growth state were sacrificed, and using the above-described method, the tumors were re-implanted and when they reached a size of 50–100 mm^3^, the tumor-bearing mice were randomly divided into five groups (six mice for each group): the control group (isometric normal saline), the low-, medium-, and high-dose VB groups (20, 40, and 80 mg/kg/day, respectively) and the fluorouracil (5-FU) group (1 mg/kg/day). VBand 5-FU were administered by tail vein injection. At days 1, 4, 7, 10, and 14, the long diameter (a) and the short diameter (b) of each tumor was measured, and tumor volume was calculated as [(a × b^2^)/2]. After 14 days of treatment, mice were sacrificed and their tumors were dissected and connective tissues were removed. The tumors were weighed. We then calculated the tumor volume inhibition rates [(1− average tumor volume of the experimental group/average tumor volume of the control group) × 100%]; and the tumor weight inhibition rates [(l − average tumor weight of the experimental group/average tumor weight of the control group) × 100%].

### Protein extraction and western blot

Western blot analyses were conducted as previously described [19, 20]. Briefly, HCT-116 cells were treated by VB (25, 50, and 100 μM) for 48 h, before being lysed and total protein was extracted. Protein samples were separated with 10%SDS-PAGE gel, transferred to a PVDF membrane with a Trans-Blot (Bio-Rad). The membrane was probed with primary antibodies (1: 1000 of anti-HIPK2, 1: 1000 of anti-P53, 1: 1000 of anti- p-p53, 1:1000 of anti-Bax, 1: 1000 of anti-Bcl-2, or 1: 4000 of anti-β-actin; Cell Signaling Technology, Danvers, MA, USA). The signal intensities of protein abundance were quantitatively analyzed by Image J.

### Statistical analysis

Software SPSS18.0 was used for statistical data analysis. The data was expressed with x ± s. If data met the homogeneity of variance of Gaussian distribution, we used one-way analysis of variance for statistical inference; otherwise, we used non-parametric tests. The test criterion *α* = 0.05, *P* < 0.05 was considered statistically significant.

## Results

### HIPK2 protein levels and CRC clinicopathologic features are inversely associated

**Figure 1:**
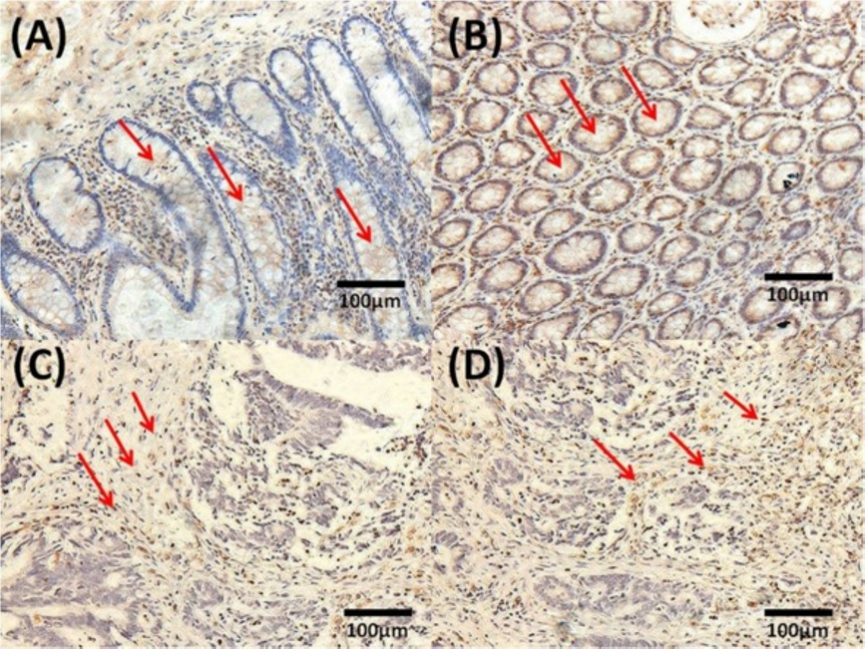
**Representative HIPK2 IHC staining in normal and colorectal tumor tissue.** Low HIPK2 protein expression in the normal tissue (−and +, Figure 1 **A**), high HIPK2 protein expression in normal tissue (++ and +++, Figure 1 **B**). Low HIPK2 protein expression in colorectal tumor tissue (−and +, Figure 1 **C**) and high expression incolorectal tumor tissue (++ and +++, Figure 1 **D**). Magnification × 200.

In 100 cases of human CRC cancer samples, 74 expressed low levels of HIPK2 protein (−and +, Figure 1A) and 26showed high expression (++ and +++, Figure 1B). In 20 cases of normal colorectal cancer tissues, eight had low HIPK2 protein expression (−and +, Figure 1C) and 12had high expression (++ and +++, Figure 1D). Expression of HIPK2 was significantly higher in normal tissues than in CRC tissues (Table 1). We further found that HIPK2 protein expression in human CRC significantly correlated with the degree of differentiation (Table 2). However, the HIPK2 expression levels were not significantly associated with sex, age, maximum tumor diameter, Dukes staging, degree of cancer infiltration, or number of metastasized lymph nodes.

**Table 1:** **Differential expression of HIPK2 in cancerous and normal colorectal tissues**

Group	***N***	Expression of HIPK2 (%)		***P***
		Low	High	
Normal colorectal tissues	20	40%	60%	0.003
Colorectal cancer tissues	100	74%	26%	

**Table 2:** **Relationship between clinicopathological parameters and HIPK2 expression in human CRC**

Variable		***N***	***N***at different HIPK2 expression levels				χ ^2^	***P***
			Low expression		High expression			
			−	+	++	+++		
Sex								
	Male	47	23	13	7	4	0.31	>0.05
	Female	53	26	12	12	3		
Age (years)								
	≤60	29	11	9	7	2	0.88	>0.05
	>60	71	38	16	12	5		
Maximum diameter of tumor								
	≤5	58	30	16	8	4	2.02	>0.05
	>5	42	19	9	11	3		
Degree of differentiation								
	Well differentiated	12	7	2	2	1	6.44	<0.05
	Moderately differentiated	67	33	16	12	6		
	Poorly differentiated	5	1	4	0	0		
Depth of invasion								
	Not invading serosa	42	28	7	5	2	4.71	>0.05
	In serosa	25	6	9	9	1		
	Outside serosa	33	15	9	5	4		
Duke stage								
	Stages A and B	72	35	19	15	3	0.13	>0.05
	Stages C and D	28	14	6	4	4		
Lymph node status								
	Metastasis	65	32	18	12	3	0.82	>0.05
	No metastasis	35	17	7	7	4		
TNM stage								
	Stages I and II	57	31	14	11	1	1.69	>0.05
	Stages III and IV	43	18	11	8	6		

### Pro-apoptotic effects of VB in CRC xenograft tumors

**Figure 2:**
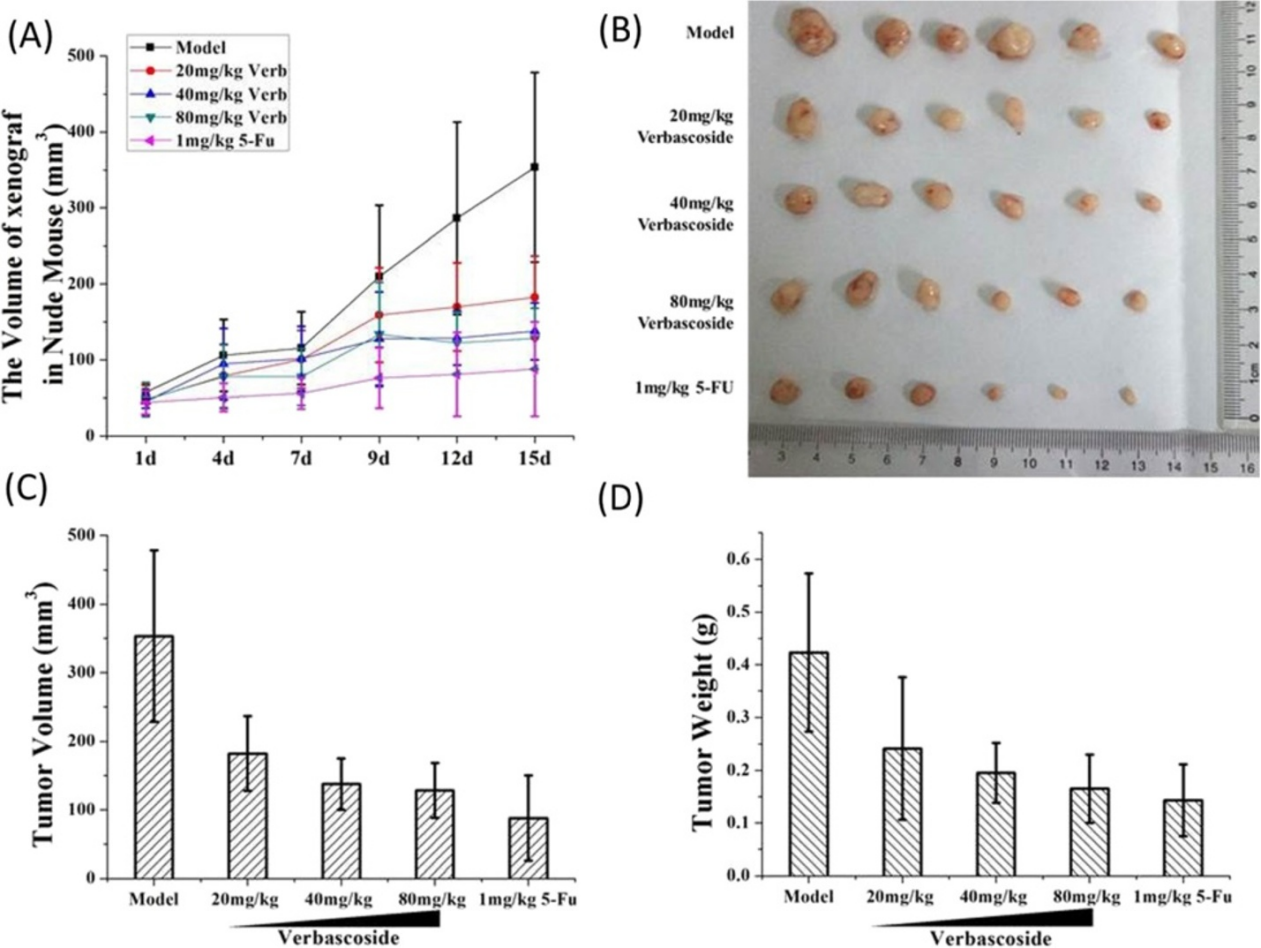
**Verbascoside (VB) inhibited** ***in vivo*** **growth of CRC tumor in a dose- and time-dependent manner.** Growth of xenograft tumors in nude mice treated with different doses of VB and 5-FU at 1, 4, 7, 9, 12, and 15 days **(A)**. Dissected tumor samples from nude mice in different treatment groups **(B)**. Xenograft tumor volumes of mice treated with indicated doses of VB and 5-FU, respectively: 353.4 ± 124.8 mm^3^, 182.4 ± 54.5 mm^3^, 137.7 ± 37.5 mm^3^, 128.1 ± 40.2 mm^3^, and87.9 ± 62.0 mm^3^ **(C)**, and xenograft tumor weights of mice treated with indicated doses of VB and 5-FU, respectively: 0.423 ± 0.150 g, 0.242 ± 0.135 g, 0.195 ± 0.057 g, 0.165 ± 0.065 g, and 0.143 ± 0.067 g **(D)**. *P* < 0.05.

**Figure 3:**
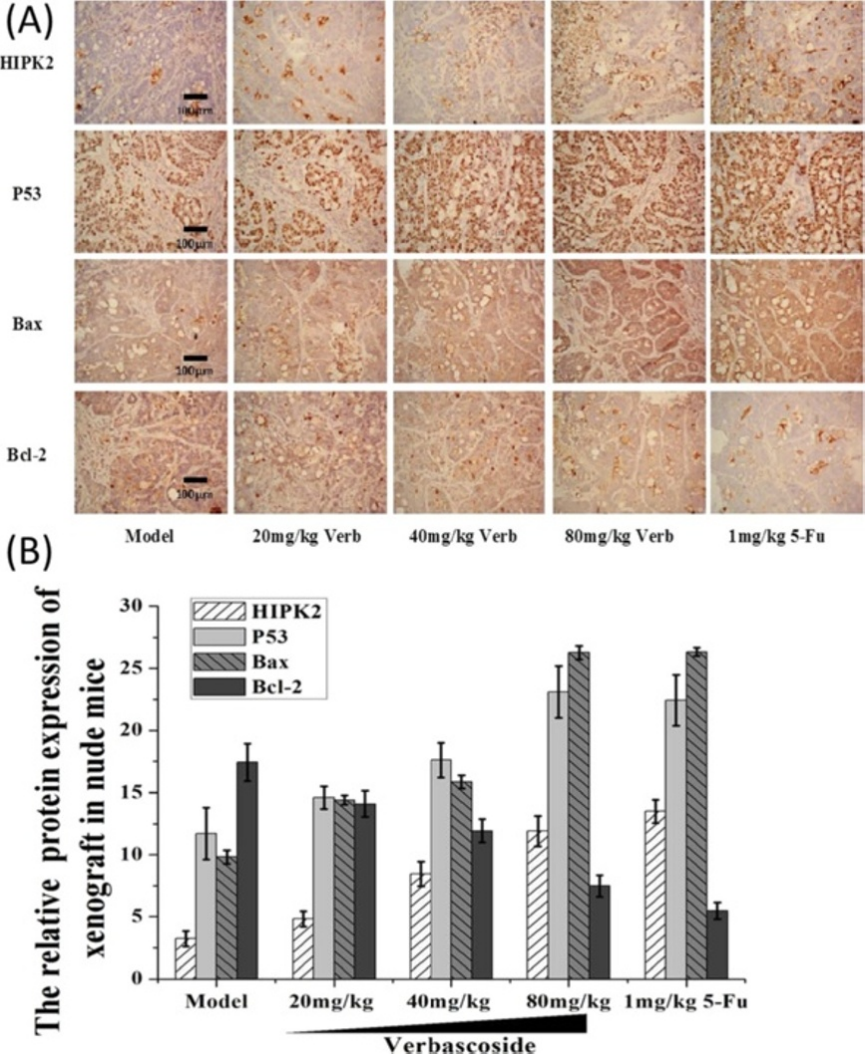
**Expression of apoptosis-related proteins is affected by Verbascoside (VB) in CRC xenograft tumors.** IHC staining of HIPK2, p53, Bax, and Bcl-2 proteins in tumors treated with VB **(A)**. Relative protein expression levels in **(A)** were quantified by image analysis software **(B)**. Magnification × 200.

To investigate the tumor inhibitory activity of VB for CRC, we first established a human CRC xenograft mode in mice, which were then treated with different doses of VB. *In vivo* data showed that VB remarkably inhibited growth of the xenografted tumors (Figure 2A and B). Tumor volume inhibition rates in the low-, medium-, and high-VB dose groups were 48.41%, 61.04%, and 63.75%, respectively; and tumor weight inhibition rates were 42.79%, 53.90%, and 60.99%, respectively (Figure 2C, D).Notably, at higher doses, the anti-tumor effect of VB was similar to that of 5-FU (Figure 2). The VB-treated tumor samples were then analyzed by IHC for levels of apoptosis-related proteins such as HIPK2, p53, Bax, and Bcl-2. The results indicated that VB significantly enhanced expression of pro-apoptotic HIPK2, p53, and Bax proteins in tumors, but decreased expression of anti-apoptotic protein Bcl-2, in a dose-dependent manner (Table 3, Figure 3).

**Table 3:** **Effect of Verbascoside on expression levels of apoptosis-related proteins in CRC xenograft tumors**

Groups	***n***	Relative protein expression level			
		HIPK2	P53	Bax	Bcl-2
Control	6	3.23 ± 0.61	11.70 ± 2.08	9.82 ± 0.55	17.43 ± 1.50
20 mg/kg VB	6	4.83 ± 0.62	14.59 ± 0.90	14.41 ± 0.38	14.08 ± 1.04
40 mg/kg VB	6	8.46 ± 0.99	17.60 ± 1.40	15.84 ± 0.54	11.93 ± 0.93
80 mg/kg VB	6	11.90 ± 1.21	23.10 ± 2.10	26.28 ± 0.55	7.48 ± 0.86
1 mg/kg 5-FU	6	13.50 ± 0.94	22.44 ± 2.05	26.34 ± 2.33	5.46 ± 0.67

### In vitro inhibitory effect of VBon CRC cells

**Figure 4:**
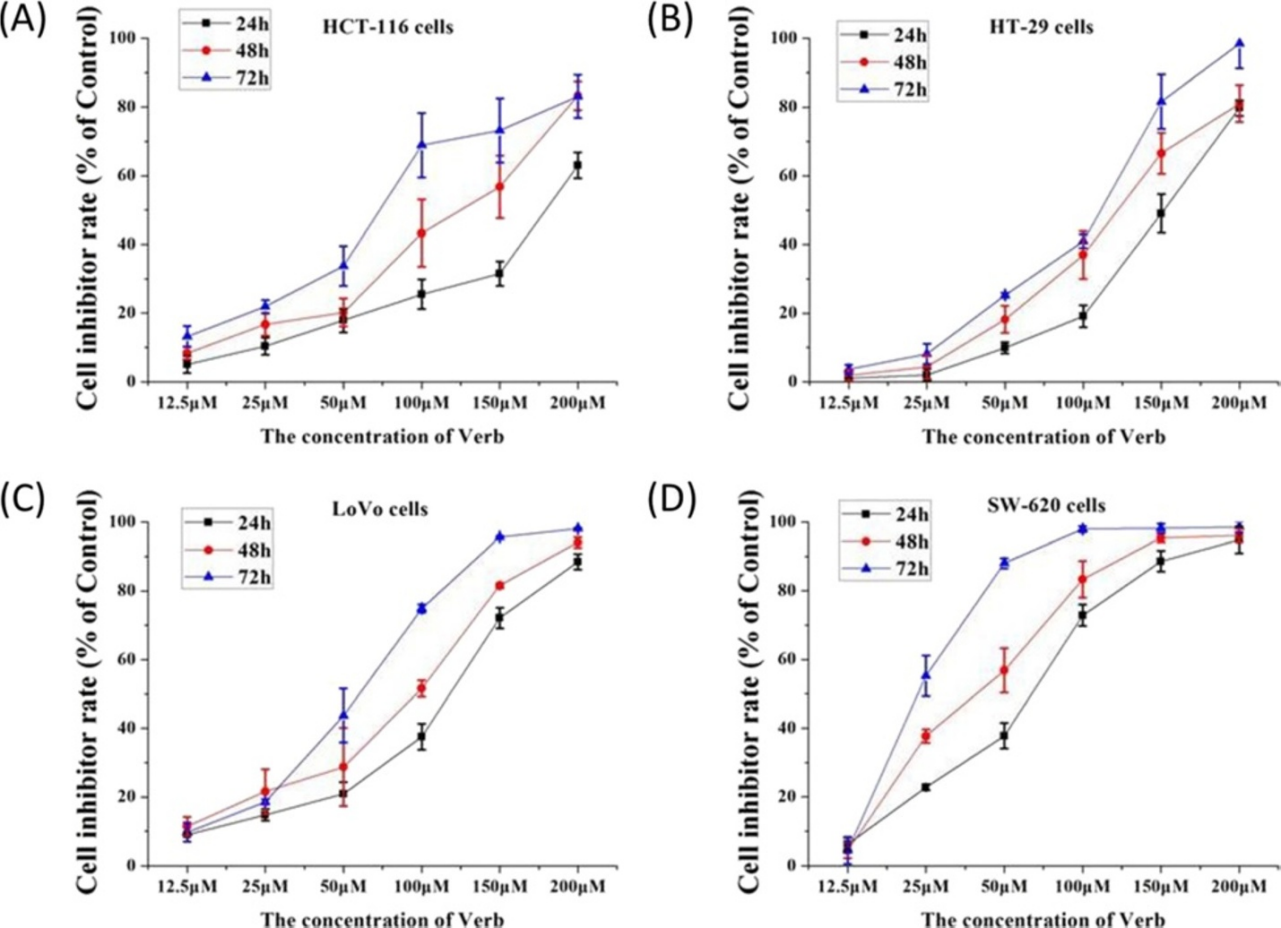
**Inhibitory effect of Verbascoside (VB) on different human CRC cell lines, at indicated doses and durations.** The IC_50_ doses of VB on human CRC cells at 24, 48, and 72-h culture, respectively, were 208.89 μmol/L, 97.86 μmol/L and 63.51 μmol/L in HCT-116 cells **(A)**; 83.83 μmol/L, 59.62 μmol/L, and 43.96 μmol/L in LoVo cells **(B)**; 144.5 μmol/L, 108.82 μmol/L, and 66.68 μmol/L in HT-29 cells **(C)**; and 52.73 μmol/L, 42.42 μmol/L, and 29.05 μmol/L in SW620 cells **(D)**.

We next tested whether VB affected *in vitro* growth of CRC cell lines. After 24, 48, and 72 h of VB treatment, the growth of CRC cells HCT-116, LoVo, HT-29, and SW620 was dramatically inhibited, in a time- and dose-dependent manner, with an IC_50_of 29–67 μM after 72 h (Figure 4).

### VB promoted apoptosis via p53 in human CRC cells

**Figure 5:**
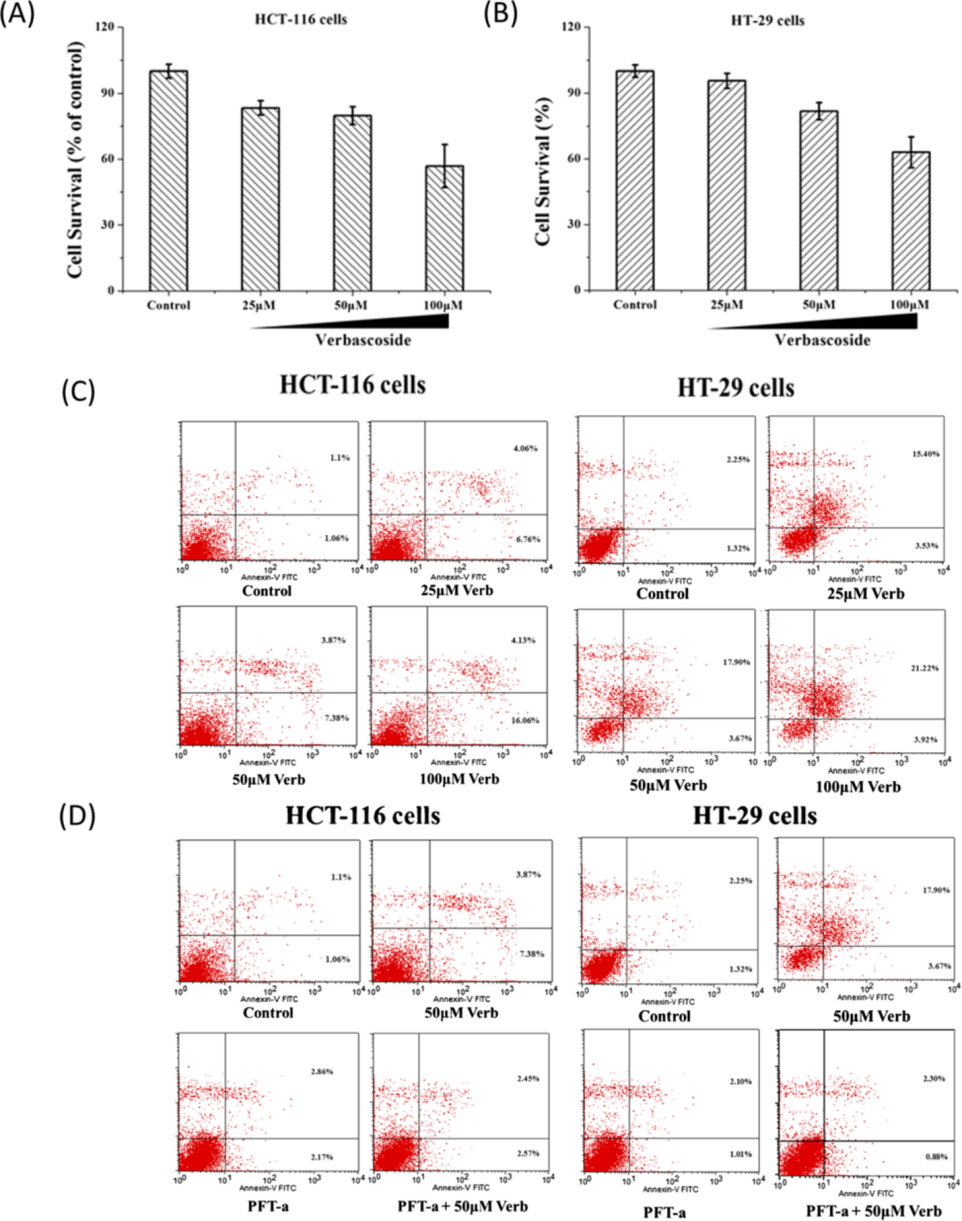
**Verbascoside (VB) promoted apoptosis via p53 in human CRC cells.** CRC HCT-116 and HT-29 cells were treated by VB at indicated doses and duration, and then analyzed for apoptosis by flow cytometry. Inhibition at 25, 50, and 100 μM of VB to HCT-116 cells was 20.20 ± 4.08%, 43.28 ± 9.80%, and 56.79 ± 9.11% **(A)**, and HT-29 cells, 4.36 ± 3.39%, 18.22 ± 3.94%, and 37.01 ± 6.98%, respectively **(B)**. HCT-116 apoptosis rate after being treated with 25, 50, and 100 μM of VB was 10.83 ± 1.28%, 11.25 ± 1.54%, and 20.19 ± 2.8%, and the HT-29 apoptosis rate, 18.92 ± 6.12%, 21.57 ± 4.05%, and 25.14 ± 6.73%, respectively **(C)**. HCT-116 and HT-29 apoptosis rate was 11.25 ± 1.54% and 21.57 ± 4.05% after being treated with 50 μM Verbascoside; and 5.03 ± 2.77% and 3.11 ± 1.16% after being treated with FPT-a (p53-specific inhibitor); and 5.02 ± 0.73% and 3.18 ± 1.82% after being treated with FPT-a and 50 μM VB respectively **(D)**.

Based on the cell proliferation inhibition data, we selected 48-htreatment of CRC HCT-116 and HT-29 as the optimal time frame for apoptosis experiments. We used drug doses of 25, 50, and 100 μM of VB to treat cells for 48 h (Figure 5A, B), and used FITC Annexin-V/PI method to measure apoptosis induced by VB. Our data showed the apoptosis rate to be significantly increased by VB in a dose-dependent manner (Figure 5C). Interestingly, this pro-apoptotic effect by VB was countered by a p53-specific inhibitor, FPT-a (Figure 5D). This suggests that VB promotes apoptosis in CRC cells through ap53-dependent mechanism.

### VB promotes apoptosis in human CRCviaHIPK2–p53signaling pathway

**Figure 6:**
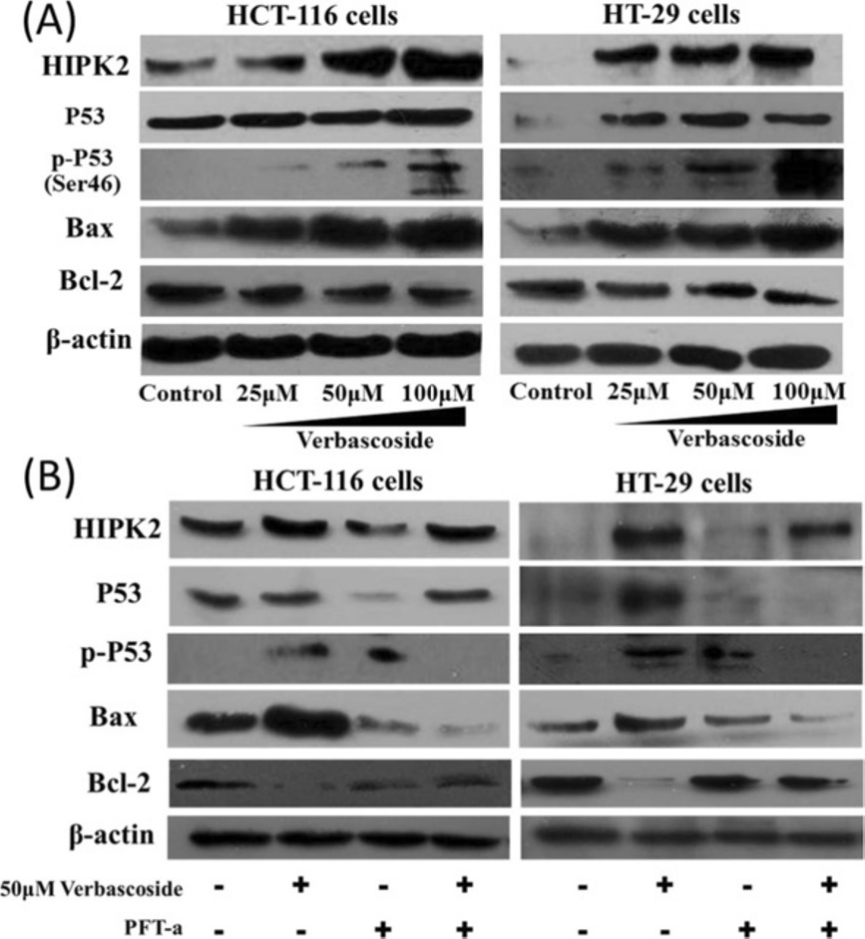
**Verbascoside (VB) alters levels of HIPK2–p53apoptosis signaling molecules in CRC cells.** HCT-116 and HT-29 cells treated with VB extracts were probed for HIPK2, p53, p-p53, Bax, and Bcl-2 protein **(A)**, and were compared with cells treated with both VB extracts and PFT-a **(B)**.

We next determined if expression levels of apoptosis-related proteins changed in VB-treated human CRC cells HCT-116 and HT-29. We found, after 48 h of treatment, VB increased protein expression of HIPK2, p53, p-p53, and Bax, but decreased that of Bcl-2, in a dose-dependent manner in the CRC cell lines (Figure 6A). These data both recapitulated the results we saw in the VB-treated CRC tumors *in vivo*, and further indicated that VB promotes apoptosis in CRC, probably through HIPK2–p53signaling axis. To verify this point, we added the p53-specific inhibitor PFT-a to the treated cells along with VB. The results showed that PFT-a rescued the cells from VB-induced apoptosis, by reducing VB-enhanced protein levels of p-p53 on Ser46, Bax, and restoring Bcl-2 protein expression, but did not affect HIPK2 protein levels (Figure 6B). These findings strongly suggest that VB-induced apoptosis is mediated by the HIPK2–p53signaling pathway.

## Discussion

Apoptosis is a response of cells to internal and external signals under certain physiological and pathological circumstances, to maintain homeostasis [21]. Many anti-cancer drugs attack tumors by triggering apoptosis [22]. Mechanisms of drug-induced tumor apoptosis include altering cell signaling pathways, expression levels of tumor-suppressor oncogene products, and influencing other apoptosis-promoting and -inhibiting proteins. Anti-cancer drugs can also block the cell cycle and inhibit cell growth, while activating caspase cascades and modulating telomerase expression and activity [23–25].

As a newly found auxiliary transcription inhibition factor, HIPK2 has been suggested to affect many aspects of cancer. Studies showed that HIPK2 participates in a variety of signal transduction pathways, including p53 [26], Wnt/β-catenin [27], JNK [28], and hypoxiainducible factor [11, 29, 30]. Recent studies suggest that HIPK2 influences apoptosis through a variety of mechanisms, especially the p53-mediated apoptosis signaling cascade [19, 20]. *p53* is the most important tumor-suppressor gene, and is implicated in regulation of apoptosis; its protein is activation is controlled by post-translational modifications, such as phosphorylation, acetylation, and interactions with other proteins. p53 phosphorylation not only stabilizes and enhances the transcription activity of p53, but also regulates its subcellular localization. p53 serine (Ser46) phosphorylation is critical to transcription of apoptosis-related genes. HIPK2 overexpression stabilizes and activatesp53 and promotes its binding to form the HIPK2–p53 complex, leading to Ser46 phosphorylation and increased apoptosis [31].

We conducted a retrospective analysis on 100 primary CRC tumor samples, and found that the average age of CRC diagnosis was 67.25 ± 11.91 years, which did not significantly vary by sex. Common symptoms of CRC include changes in bowel habits, hemafecia/melena, and abdominal pain or discomfort. Among them, hemafecia is the most common symptom, seen in 93.75% of patients with CRC. As for the clinicopathological features, the average tumor diameter was 5.31 ± 2.21 cm, with glandular cancer as the most common histology (91%), and ulcerative type as the major morphological type (37%). IHC analyses showed HIPK2 expression in normal colorectal mucosal tissues to be higher than in CRC samples. These data are consistent with previous reports showing a similar pattern for HIPK2 expressions in breast cancer and thyroid cancer [32–34]. Correlation analysis showed that HIPK2 expression was closely associated with Dukes staging and infiltration degrees, but not to sex, age, degree of differentiation, or lymph node metastasis.

We next tested VB’s anti-tumor activity in an *in vivo* mouse model of human CRC, and found VB to significantly inhibit xenograft tumor growth. IHC analyses showed heightened levels of pro-apoptotic proteins HIPK2, p53, Bax, and decreasedBcl-2 in VB-treated tumors. These results imply that VB promotes cancer cellapoptosis throughHIPK-2- and p53-related signaling. To study the mechanisms of this anti-cancer effect, we used VB to treat human CRC cell lines. As with the *in vivo* studies, VB had a remarkable anti-proliferative and apoptosis-promoting effect in HCT-116, HT-29, LoVo, and SW620 cells, in a time- and dose-dependent manner. In addition, this nicely correlates with the previous finding that VB induces genotoxic stress [35].

Reportedly, theHIPK2–p53 apoptotic pathway is downregulated in different human cancer cells [36–42]. In investigating the mechanisms that underpin VB-promoted apoptosis, we first learned that both in CRC tumors and cells, VB elevated HIPK2 protein levels. Additionally, levels of p53, p-p53 at Ser46, and downstream pro-apoptosis Bax protein were greatly boosted, whereas anti-apoptosis Bcl-2 protein expression was reduced, by VB treatment. Furthermore, the pro-apoptotic action of VB was obscured by a p53-specific inhibitor, which restored protein levels of p-p53 (Ser46), p53, Bax, and Bcl-2 to the untreated status. Interestingly, HIPK2 protein expression was not influenced. To summarize, our data suggest that VB promotes p53 phosphorylation and Bax expression and inhibits Bcl-2 expression by increasing HIPK2 levels in CRC, which leads to activation of theHIPK2–p53 signaling pathway and increased apoptosis.

## Conclusions

In summary, we found that HIPK2 expression inversely correlates with primary CRC, Dukes staging, and infiltration degrees. We also found that VB significantly inhibits CRC growth *in vivo*, and represses CRC cell proliferation, and promotes apoptosis, by modulating the HIPK2–p53 signaling pathway.
